# Distinct Functional and Temporal Requirements for Zebrafish *Hdac1* during Neural Crest-Derived Craniofacial and Peripheral Neuron Development

**DOI:** 10.1371/journal.pone.0063218

**Published:** 2013-05-07

**Authors:** Myron S. Ignatius, Arife Unal Eroglu, Smitha Malireddy, Glen Gallagher, Roopa M. Nambiar, Paul D. Henion

**Affiliations:** 1 Department of Neuroscience, Ohio State University, Columbus, Ohio, United States of America; 2 Molecular, Cellular and Developmental Biology Program, Ohio State University, Columbus, Ohio, United States of America; University of Sheffield, United Kingdom

## Abstract

The regulation of gene expression is accomplished by both genetic and epigenetic means and is required for the precise control of the development of the neural crest. In *hdac1^b382^* mutants, craniofacial cartilage development is defective in two distinct ways. First, fewer *hoxb3a, dlx2* and *dlx3-*expressing posterior branchial arch precursors are specified and many of those that are consequently undergo apoptosis. Second, in contrast, normal numbers of progenitors are present in the anterior mandibular and hyoid arches, but chondrocyte precursors fail to terminally differentiate. In the peripheral nervous system, there is a disruption of enteric, DRG and sympathetic neuron differentiation in *hdac1^b382^* mutants compared to wildtype embryos. Specifically, enteric and DRG-precursors differentiate into neurons in the anterior gut and trunk respectively, while enteric and DRG neurons are rarely present in the posterior gut and tail. Sympathetic neuron precursors are specified in *hdac1^b382^* mutants and they undergo generic neuronal differentiation but fail to undergo noradrenergic differentiation. Using the HDAC inhibitor TSA, we isolated enzyme activity and temporal requirements for HDAC function that reproduce *hdac1^b382^* defects in craniofacial and sympathetic neuron development. Our study reveals distinct functional and temporal requirements for zebrafish *hdac1* during neural crest-derived craniofacial and peripheral neuron development.

## Introduction

The neural crest is a transient embryonic cell population that gives rise to craniofacial cartilages, neurons and glia of the peripheral nervous system and pigment cells, among other cell types [1]. Multiple regulators of gene expression required for neural crest development have been identified. These include the Bmp, Fgf, Notch and Wnt signaling pathways [2]–[4] and key transcription factors including *foxd3* [5]–[8], *tfap2a* [9]–[11], *snail1b* [12], [13], *pax3* [14], *sox9* [15], [16] and *sox10* [17], [18]. Disruptions in several of these genes have been shown to result in neuropathies, pigmentation and craniofacial defects in humans [19]. In eukaryotic cells, gene expression can also be regulated by control of chromatin structure [20], [21]. Chromatin consists of DNA associated with histone and non-histone proteins. The basic unit of chromatin is the nucleosome, which is is composed of 146 bp of negatively charged DNA wrapped around an octameric core of positively charged histone proteins. Numerous studies have implicated specific modifications in the histone tails or combinations of epigenetic modifications as being important for regulating gene transcription [22]–[24]. For example, the activation or repression of gene expression correlates with the acetylation state of histones [25]. In general, acetylated histones are associated with more open chromatin and correspondingly active gene expression, whereas deacetylated histones are usually associated with closed chromatin and repressed gene expression [26]. The acetylation status of histones within eukaryotic cells is catalyzed by two types of enzymes known as histone deacetylases (HDACs) and histone acetyltransferases (HATs) [27]–[29]. HDAC enzymes are classified into four classes based on homology studies [30].

While the global effects of histone acetylation on gene expression are recognized, the requirements for individual HDACs are presently not well understood. Furthermore, tissue specific requirements for individual HDACs are just being discovered. *hdac1* encodes a Class 1 HDAC that so far has been shown to be required in multiple tissues and molecular pathways in zebrafish and mice. In zebrafish for instance, *hdac1* is required for aspects of the development of the eye, CNS and neural crest cell populations [31]–[35]. Zebrafish *hdac1* has been found to be a negative regulator of the canonical Wnt signaling pathway during early dorsal-ventral patterning and subsequent anterior-posterior patterning of the brain [33]. *hdac1*-mediated regulation of the canonical Wnt signaling pathway is also required to control the proliferation of retinal precursors [35]. *hdac1* is not only a negative regulator of the canonical Wnt signaling pathway but is also a positive regulator of the non-canonical Wnt signaling pathway that is required for anterior-posterior axis elongation in zebrafish [36]. In addition to being a regulator of the Wnt signaling pathway, *hdac1* is a negative regulator of Notch signaling in the developing zebrafish retina and CNS [31]. During early neural crest development, *hdac1* is required to specify melanophores by negatively regulating the expression of *foxd3* in nascent melanogenic progenitors [32]. *hdac1* has also been shown to be required for normal craniofacial and fin development in zebrafish [33], [37], yet the developmental processes requiring *hdac1* function have not previously been determined.

An important finding related to *hdac1* function in zebrafish is that the requirement for *hdac1* for development is not uniform but appears to be both cell type and tissue specific. In the retina for instance, *hdac1* functions as a switch from proliferation towards differentiation [34], [35]. In contrast, in the hindbrain, *hdac1* is required for cell proliferation [31]. In mice, Hdac1 knock out mutants die at E9.5, potentially due to proliferation deficits [38]. A conditional viable mouse knockout has identified a cardiac-specific role for Hdac1 that is manifested only when Hdac1 and Hdac2 are both eliminated in the heart [39]. Given the recent interest in HDAC inhibitors as potential drug candidates in the treatment of cancer, degenerative diseases and effects on learning and memory [40], [41], it is necessary to study the functions of individual HDACs in multiple contexts to fully understand their roles in development as well as in disease conditions.

In this study, we determined the functions of *hdac1* during neural crest-derived craniofacial and peripheral neuron development utilizing *hdac1^b382^* mutants and the pharmacological HDAC inhibitor trichostatin A (TSA). We find that *hdac1* is required reiteratively yet differentially during craniofacial and peripheral neuron development. In craniofacial development, defects observed are in part due to early cell fate specification deficits in posterior branchial arch precursors in *hdac1^b382^* mutants. In contrast, abnormal development of the anterior mandibular and hyoid arches in *hdac1^b382^* mutants are due to later differentiation defects. Neural crest-derived peripheral neuron development is also abnormal in *hdac1^b382^* mutants. In enteric and DRG neurons, there is a pronounced disruption in the anterior-posterior patterning of neurons and ganglia as well as a delay in neuronal differentiation in *hdac1^b382^* mutants. During sympathetic neuron development, sympathoblasts are specified normally and undergo generic neuronal differentiation in *hdac1^b382^* mutants but noradrenergic differentiation fails to occur. Interestingly, we found that the prevention of noradrenergic differentiation of sympathetic neurons by the disruption of HDAC function is reversible, illustrating a significant temporal plasticity in this process during development. Taken together, our results demonstrate surprising degrees of both cell type- and developmental mechanism-specific requirements for *hdac1* during neural crest development in zebrafish.

## Materials and Methods

### Animal Husbandry and Genotyping

Adult zebrafish and embryos were raised and maintained at 28.5°C in the Ohio State University Zebrafish facility. *hdac1^b382^* mutant embryos were obtained by pair wise matings of heterozygous adult zebrafish that were maintained in AB and WIK backgrounds. Embryos were staged according to Kimmel et al. (1995) [42]. Two methods were employed to determine the genotype of embryos before a visible phenotype was clearly apparent. Genomic DNA from individual embryos was obtained and PCR was performed using chromosome 19-linked polymorphic SSLP markers that have been shown to be closely linked to the *col* locus [33], [43], [44]. The other method used for genotyping is specific for the *hdac1^b382^* mutant allele. The *hdac1^b382^* mutation is a T-to-G transversion in the intron sequence flanking exon 5 that creates a new splice acceptor site resulting in the addition of 9 bp from adjacent intron 4 to exon 5 [36]. The T-to-G transversion creates a new EcoR1 restriction site that can be used to identify mutants from wild-type and heterozygous embryos when intron 4 is PCR amplified using primers (F-5′-TAACGTAGGGGAGGATTGTC-3′, R-5′-CAGCTCCAGAATGGCCAGTAC-3′). The PCR product obtained when treated with EcoR1 cleaves the mutant but not wild-type DNA and both DNA products can be resolved on a 2% agarose gel.

### Alcian Blue Staining

Alcian blue staining was performed according to Schilling et al. (1994) [45] with the following modifications. Embryos were treated with 0.05–0.1% of alcian blue for 6–8 h. To clear stained cartilages, embryos were treated with 0.5% of lyophilized pancreatic lysate, obtained from Sigma, instead of trypsin. The pancreatic lysate was dissolved in a saturated sodium tetrahydroborate solution.

### In Situ Hybridization, Immunohistochemistry, Cell Death Assays, Western, Statistical and Image Analyses

In situ hybridization was carried out on staged embryos as described previously [46] with minor modifications. Embryos older than 24 hpf were raised in 0.03g/l 1-phenyl-2- thiourea (PTU) to prevent melanin synthesis. Probes used were *crestin* [47], [48], *col2a1* [49], *dbh* [50], *dlx2* [51, *dlx3* [51, *hand2* [52], *hoxb2a* [53], *hoxb3a* [53], *phox2a* [54], *phox2b* (J. Holzchuh, University of Freiburg, Germany; provided by Dr. S. Guo), *sox9a* [15], *sox10* [17], *tbx1* [55], *th* [56], *zash1a* [8].

Immunohistochemistry was performed on sections and whole embryos fixed in 4% paraformaldehyde for 2 h at room temperature and performed according to Henion et al., 1996. The 16A11 antibody was obtained from Invitrogen. Apoptotic cell death was detected in whole embryos by terminal transferase dUTP nick-end labeling (TUNEL) using modifications suggested by the manufacturer (In Situ Cell Death Detection Kit; Roche).

Protein extracts from five zebrafish wildtype or *hdac1^b382^* embryos at each time point were dissolved in blending buffer (10% SDS, 62.5 mM Tris pH6.8, 5 mM EDTA) and then resolved in a 12% bis-Tris gel, electro-blotted to a polyvinylidene difluoride membrane (Hybond-P; Amersham Bioscience). Membranes were probed with primary antibodies: anti-Hdac1 (1∶500, ABCAM, ab33278), ß-actin (1∶5000, Santa Cruz, sc-47778). Signal was detected with horseradish peroxidase-conjugated goat anti-mouse antibodies (1∶5000, Jackson ImmunoResearch Laboratories, Inc.) and ECL reagents (Amersham Bioscience).

Graph prism pad version 4.0 software was used to conduct statistical analysis. Embryos were examined on a Leica MZ16 stereomicroscope and a Zeiss axioplan compound light and fluorescence microscope, and figures were assembled using Photoshop CS2. In some figure panels, to analyze complete embryos, images of embryos that were captured in the same positions but at slightly different focal planes were fused together using Photoshop CS2.

### TSA Treatment

Trichostatin A (TSA, Sigma) was dissolved in DMSO at a concentration of 1 mg/ml. This stock solution was then diluted in embryo medium to 50, 100, 400, 600 and 800 nM concentrations. TSA solutions added to embryos were changed every 12 hours and after treatment, embryos were washed 3 times in excess embryo medium to remove TSA. Washed embryos were allowed to develop in embryo medium to required stages and fixed in 4% paraformaldehyde. For experiments involving sympathetic neurons, embryos were treated with TSA diluted in embryo medium containing 0.03 g/l 1-phenyl-2-thiourea (PTU) to prevent melanin synthesis.

## Results

### Differential and Asynchronous Craniofacial Neural Crest Developmental Defects in *hdac1^b382^* Mutants

**Figure 1:**
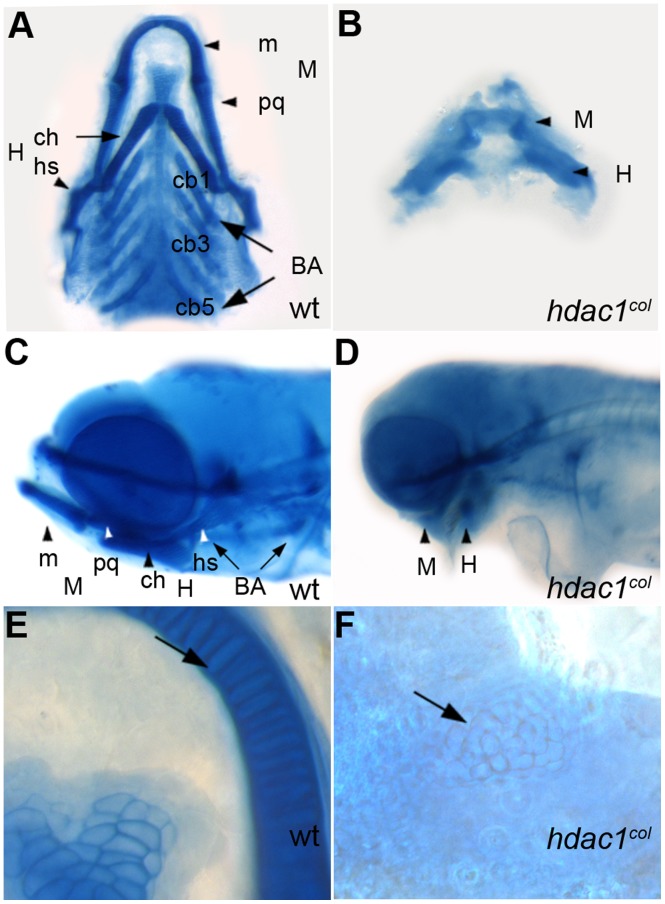
Craniofacial defects in *hdac1^b382^* mutants. A–E alcian blue stained jaw elements in 5 dpf wild type A, C, E and *hdac1^b382^* mutants B, D, F; A, B Ventral view of dissected craniofacial cartilages of wild-type *and hdac1^b382^* mutant; C, D lateral view of head region in wild-type and *hdac1^b382^* mutant; E,F, High magnification of the mandibular chondrocytes (arrows) in wild-type and *hdac1^b382^* mutant; m, meckels; pq, platoquadrate; M, mandibular; ch, ceratohyal; hs, hyosymplectic; H, hyoid; cb1-5, ceratobrachials 1-5; BA, branchial arches.

In wildtype embryos at three days post fertilization (3 dpf), neural crest-derived cells of the pharyngeal arches differentiate and condense into distinct cartilage producing elements of the jaws detectable by alcian blue staining. Similarly, the precursor cells of the future neurocranium condense into clearly identifiable cartilaginous elements. In contrast, in *hdac1^b382^* mutants at 3 dpf there is a complete lack of alcian blue stained-cartilage elements in the jaw [33]. Also, in *hdac1^b382^* mutants the neurocranium cartilages are stained by alcian blue, however the neurocranium itself is reduced in size and the ethmoid plate elements fail to fuse at the midline as it does wildtype embryos [33]. Later, at 5 dpf, the mandibular and hyoid arches in *hdac1^b382^* mutants are very faintly stained by alcian blue, compared to wildtype embryos, however these arch elements are not easily distinguishable and are drastically reduced in size (Fig. 1 A–D). Higher magnification resolution of chondrocytes in the mandibular and hyoid arches in wildtype embryos reveals well-defined, elongated cells. In contrast, in *hdac1^b382^* mutants, few defined chondrocytes are visible and those that are observed are round in shape suggesting defects in terminal differentiation (Fig. 1 E, F). In *hdac1^b382^* mutants, the different differentiated posterior branchial arch elements are altogether absent in alcian blue stained embryos (Fig. 1 A–D) at all stages examined. Thus, the disruption of *hdac1* function in *hdac1^b382^* mutants results in early defects in the establishment of posterior pharyngeal arch neural crest progenitor populations and a later defect in anterior arch morphogenesis and differentiation.

### Defects in Mandibular and Hyoid Arch Development in *hdac1^b382^* Mutants

Previous analysis of early neural crest development revealed that neural crest cell induction is unaffected in *hdac1^b382^* mutants [32]. For instance, there is no obvious difference in the expression or numbers of *crestin* and *sox10*-positive cranial neural crest cells between *hdac1^b382^* mutants and wildtype embryos at the 6 somite stage (s), 15 s, and 25 hours post fertilization (hpf) [32]. Therefore, observed abnormalities in craniofacial cartilages in *hdac1^b382^* mutants result from later defects in cranial neural crest cell development.

**Figure 2:**
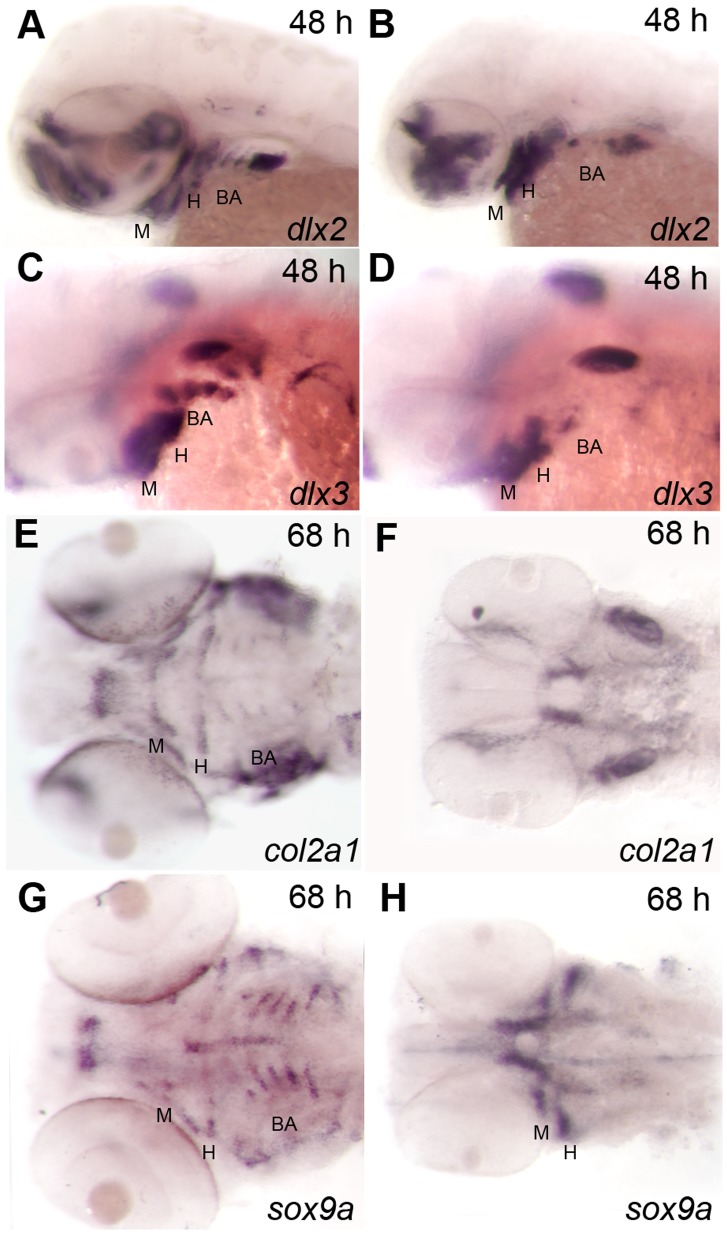
Craniofacial progenitor differentiation defects in the mandibular and hyoid arches of *hdac1^b382^*mutants. A, C, E, G wild-type, B, D, F, H *hdac1^b382^* mutants; A, B Lateral views of the head with *dlx2* expression labeling developing jaw elements in 48 hpf embryos. C, D, Lateral views of the head with *dlx3* expression labeling developing jaw elements in 48 hpf embryos. E, F ventral views of the head in 68 hpf embryos expressing *col2a1* in different jaw structures, *col2a1*. G, H ventral views of the head of 68 hpf embryos expressing *sox9a* in different jaw elements. M, mandibular; H, hyoid; BA, branchial arches.

**Figure 3:**
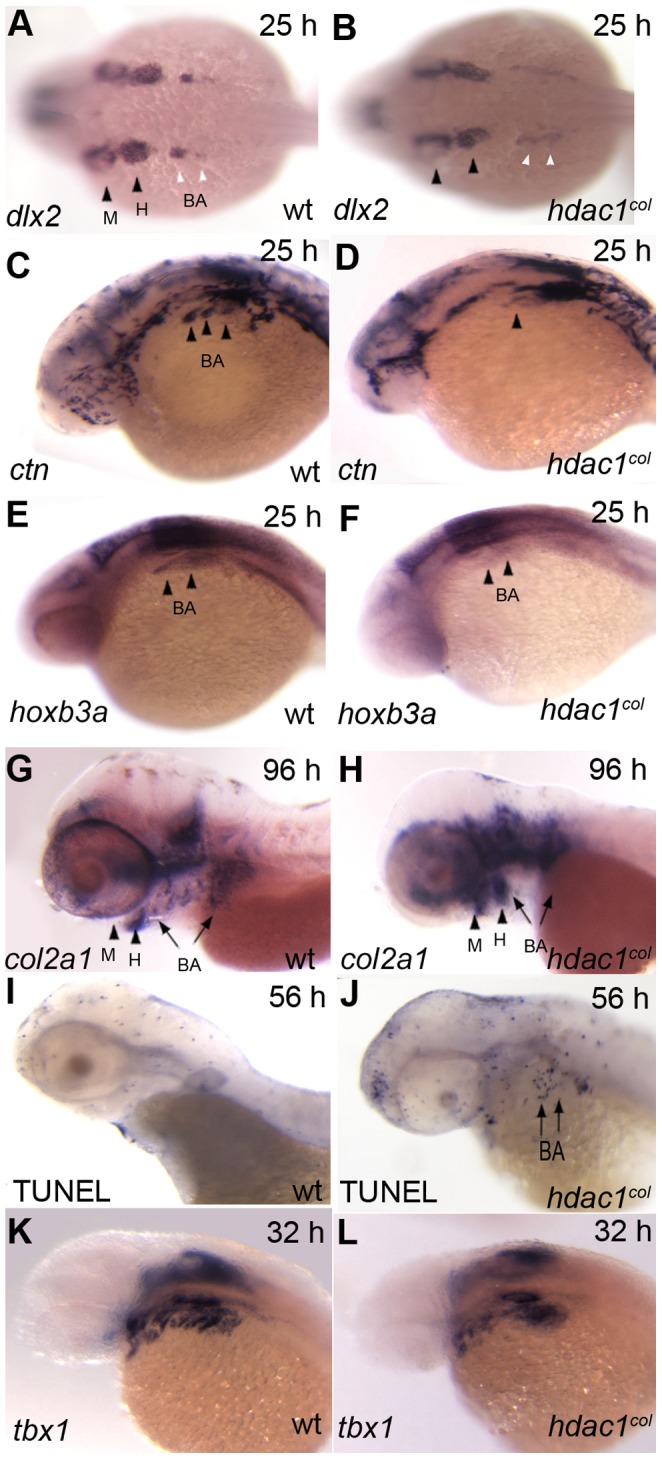
Neural crest-derived posterior branchial arch progenitor specification is defective in *hdac1^b382^* mutants. A, C, E, G, I, K wild-type embryos, B, D, F, H, J, L *hdac1^b382^* mutants. A, B Dorsal view of embryos at 25 hpf with *dlx2* expression labeling the mandibular, hyoid and branchial arch precursor populations. C, D Lateral views of the head region at 25 hpf of *crestin* expression in the head region, black arrowheads indicate branchial arch populations. E, F, Lateral views of the head region at 25 hpf of *hoxb3a* expression in the hind-brain and branchial arch precursors. G, H, Lateral views of the head region at 96 hpf of *col2a1* expression in mandibular, hyoid and branchial arches. I, J, Lateral views of the head region at 56 hpf of TUNEL-positive staining in the head and jaw regions. K, L, Side views of the head region at 32 hpf with *tbx1* expression highlighting the endodermal pouches. M, mandibular; H, hyoid; BA, branchial arches.

At 48 hpf, the pharyngeal skeleton has a distinct dorso-ventral dimension as a result of the ventral migration of neural crest-derived cells into the pharyngeal pouches [57]. Migrating pharyngeal arch neural crest cells express *dlx2*. In the mandibular and hyoid arch, while qualitatively there are equivalent numbers of migrating neural crest cells in *hdac1^b382^* mutant and wildtype embryos, cranial neural crest morphogenesis is defective in mutant embryos (Fig. 2 A, B). Consistent with defects in morphogenesis, the number of *dlx3*-positive cranial neural crest cells localized to the ventral most jaw elements, are significantly reduced in *hdac1^b382^* mutants (Fig. 2 C, D). We next analyzed the expression of *col2a1* which is a major collagen produced in the differentiating pharyngeal arch elements [15], [58]. In wild-type embryos by 68 hpf, *col2a1* is robustly expressed by all elements of the pharyngeal arches. In contrast, in *hdac1^b382^* mutants, there is a delay in the expression of *col2a1* by a day in the mandibular and hyoid arches (Fig. 2 E, F; Fig. 3 G, H). Further, there is a distinct patterning defect whereby the elements of the hyoid arch from both sides of the head fail to meet ventrally close to the middle of the head. There are also defects in the size and formation of the mandibular arch elements (data not shown).

*sox9* is required for the differentiation of cartilages in part by controlling the expression of collagens, like *col2a1*, that are required for cartilage extra-cellular matrix formation [16], [59]–[63]. In zebrafish, the ancestral *sox9* gene has undergone a gene duplication event with the function of *sox9* shared between *sox9a* and *sox9b* co-orthologs [16]. *sox9a* is expressed robustly in the mandibular and hyoid arches in wildtype and *hdac1^b382^* embryos at 48 and 68 hpf. However, like *col2a1* expression, *sox9a*-expressing mandibular and hyoid arches have easily noticeable morphological defects in *hdac1^b382^* mutants when compared to wild-type embryos (Fig. 2 G, H; data not shown). Together, the analysis of *dlx2, dlx3, col2a1*, *sox9a* and alcian blue staining data indicates that there is a late differentiation and patterning defect between 2 and 4 dpf in the anterior mandibular and hyoid arches in *hdac1^b382^* mutants.

### Neural Crest-derived Posterior Branchial Arch Progenitor Specification is Defective in *hdac1^b382^* Mutants

At 24–25 hpf in wildtype embryos, *dlx2* is expressed in four out of the eventual seven pharyngeal arch populations [51], namely the mandibular or first pharyngeal arch, the hyoid or second pharyngeal arch and the third and fourth posterior branchial arch cell-populations. In *hdac1^b382^* mutants, *dlx2*-positive cell numbers in the mandibular and hyoid arches is similar to wildtype embryos (Fig. 3 A, B). In contrast, in *hdac1^b382^* mutants, fewer *dlx2-* positive neural crest cells are present in the branchial arches. Also, the *dlx2*-positive branchial arch precursors in *hdac1^b382l^* mutants are mislocalized to a more posterior position when compared to wildtype embryos, and the cells fail to coalesce into distinct groups of cells (Fig. 3 A, B). Similar to *dlx2*, *crestin* is expressed in migrating neural crest cells that populate the pharyngeal arches at 25 hpf [47]. In *hdac1^b382^* mutants, there are reduced numbers of *crestin*-expressing cranial neural crest cells migrating into the third and fourth pharyngeal arches (Fig. 3 C, D).

Neural crest cells that form the mandibular arch do not express or require *hox* gene expression for their development. In contrast, neural crest cells migrating into the hyoid arch require *hox* group 2 genes, and the branchial arches require *hox* group 3 genes, respectively, for proper development [53]. In *hdac1^b382^* mutants, while there is only a slight reduction in *hoxb2a* cell numbers in the hyoid arch compared to wildtype embryos (data not shown), the numbers of *hoxb3a-*positive branchial arch precursors is significantly reduced at 25 hpf (Fig. 3 E, F).

The *dlx2, crestin* and *hoxb3a* expression results indicate that branchial arch specification is disrupted in *hdac1^b382^* mutants. In *hdac1^b382^* mutants, differentiated branchial arch cartilages detected by alcian blue staining are never observed (Fig. 1 A–D). In addition, *sox9a* and *col2a1* are never expressed in the branchial arches of *hdac1^b382^* mutants (Fig. 2 E–H and Fig. 3 G, H) indicating disruption of the differentiation of these cranial neural crest subpopulations. A few *crestin*-positive branchial arch cells persist at 48 hpf in *hdac1^b382^* mutants, but these cells appear to undergo cell death as suggested by increased numbers of TUNEL^+^ cells in the region (Fig. 3 I, J) between 48 and 60 hpf, the time at which *crestin* expression is normally extinguished in the branchial arches (Fig. 3 I, J).

Craniofacial patterning requires interactions between neural crest cells and mesendoderm tissues. We therefore analyzed *tbx1* expression, which is required for pharyngeal mesendoderm development [55]. At 32 hpf, there is no dramatic overall difference in the number of *tbx1*-expressing pharyngeal pouch endodermal cells between *hdac1^b382^* mutants and wildtype embryos (Fig. 3 K, L). The only obvious difference observed is a disruption in the organization of the pharyngeal pouches in the posterior branchial arch region in *hdac1^b382^* mutants when compared to wild-type embryos. This defect could potentially be due to earlier defects in branchial arch neural crest development that we have observed. Nevertheless, the possibility also exists that hdac1 functions cell autonomously in *tbx1*-expressing mesendodermal cells that may in turn have consequences for neural crest development. Taken together, the *dlx2, hoxb2a* and *hoxb3a* expression data indicate that the pattern of mandibular and hyoid arch neural crest specification is normal in *hdac1^b382^* mutants, however there is a reduction in the number of branchial arch cranial neural crest cells specified.

### The HDAC Inhibitor TSA can Reproduce the *hdac1^b382^* Mutant Cranial Neural Crest Phenotypes in Wildtype Embryos

Trichostatin A (TSA), a fungal metabolite, is a potent, reversible inhibitor of both Class I and Class II HDACs, including *hdac1*, and has been used extensively to selectively block HDAC enzyme activity both in vitro and in vivo [40]. The addition of a range of concentrations between 50 and 1200 nM of TSA to zebrafish embryos at different developmental stages, can reproduce aspects of eye, brain and the overall patterning of the body plan of *hdac1* mutants [35], [36]. We tested whether the addition of the HDAC inhibitor TSA to wildtype embryos recapitulates the cranial neural crest defects observed in *hdac1^b382^* mutants. Specifically, we tested the effects of TSA on the specification of branchial arch precursors and the morphogenesis and differentiation of mandibular and hyoid elements.

**Figure 4:**
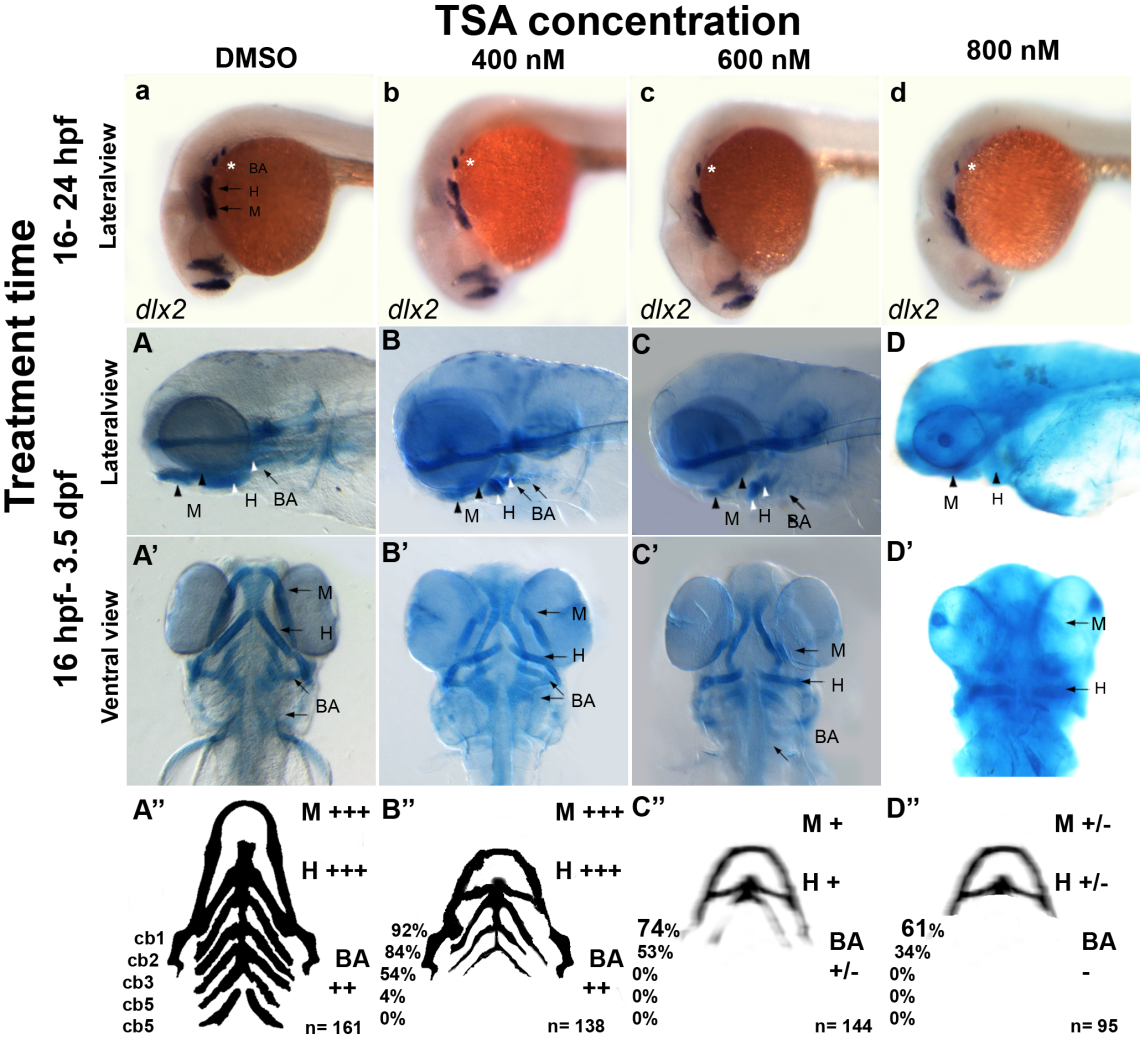
Treatment with the HDAC inhibitor TSA can reproduce the *hdac1^b382^* mutant phenotype. A–d Lateral views of wild-type embryos treated with DMSO, 400 nM, 600 nM and 800 nM TSA from 16–24 hpf after which embryos were fixed and stained for *dlx2* expression. A–D and A’–D’ Alcian blue stained 3.5 dpf wild-type embryos under different TSA treatment conditions, all embryos were treated between 16 hpf and 3.5 dpf after which embryos were fixed and then stained with alcian blue; A–A’ DMSO controls, B–B’ 400 nM TSA, C–C’ 600 nM TSA, D–D’ 800 nM TSA. A–D lateral view; A’–D’ ventral views. A’’–D’’ schematic with summary of craniofacial defects at different TSA treatment conditions. M, mandibular; H, hyoid; cb1-5, cerato-branchials 1-5; BA, branchial arches,+++ wild-type,++ reduced in size compared to wild-type,+severely reduced compared to wild-type, +/− severely reduced or absent altogether.

We added 800 nM of TSA to wildtype embryos between 16 and 24 hpf, after which embryos were fixed at 24 hpf and processed for *dlx2*-expression. We found qualitatively reduced numbers of *dlx2*-positive branchial arch precursors present in 800 nM TSA treated embryos (Fig. 4 A–D; Table 1). The 800 nM dose of TSA treatment had no effect on the numbers of *dlx2-*postive mandibular and hyoid arch-precursors present when compared to control DMSO-treated wildtype embryos. The selective effect of 800 nM TSA on neural crest branchial arch precursor specification in treated wildtype embryos, compared to mandibular and hyoid progenitors, was equivalent to that observed in *hdac1^b382^* mutants.

**Table 1:** Effect of 400, 600 and 800 nM of TSA on craniofacial development.

	16 hpf- 3.5 dpf			
	800 nM	600 nM	400 nM	Control
Mandibular	+/−	+	+	+++
%	58/95 = 61.05%	100%	100%	100%
Hyoid	+/−	+	+	+++
%	32/95 = 33.6%	100%	100%	100%
CB1	–	+	+	+++
%	0%	73.61%	92.03%	100%
CB2	–	+	+	+++
%	0%	52.77%	84.06%	100%
CB3	–	+/−	+/−	+++
%	0%	0%	53.62%	100%
CB4	–	–	–	+++
%	0%	0%	4.35%	100%
CB5	–	–	–	+++
%	0%	0%	0%	92.55%
Shape of elements	+	+	+	+++
N	95	144	138	161

Embryos were assigned the following groups based on the severity of craniofacial malformations when compared to wild-type embryos;+++ wild-type, +++/− Mild malformation,++ Moderate malformation,+Severe malformation, - Jaw elements absent or not stained.

Percentages reflect proportion of embryos with alcian blue stained cartilage element to the total number of treated embryos expressed as a percentage.

In *hdac1^b382^* mutants, there is an overall absence or severe reduction of alcian blue staining of jaw cartilages at 3.5 and 5 dpf. Similarly, the addition of 800 nM of TSA to wildtype embryos from 16 hpf until 3.5 dpf, after which treated embryos were fixed and stained with alcian blue, resulted in the absence of all alcian blue stained craniofacial cartilages in TSA-treated embryos (Fig. 4 D, D’, D”; Table 1).

At lower TSA doses of 400 and 600 nM, and similar 16 hpf to 3.5 dpf treatments, alcian blue stained craniofacial cartilages were always observed in TSA-treated wildtype embryos. However, there was a noticeable reduction in the size of the mandibular, hyoid and branchial arch cartilages, when compared to control embryos. The severity of the craniofacial cartilage reductions observed was affected by the concentration of TSA applied. Correspondingly, wildtype embryos treated with 600 nM TSA have more severe reductions in the sizes of the mandibular, hyoid and branchial arches compared to 400 nM TSA-treated embryos (Fig. 4 B, B’, B”, C, C’, C”; Table 1).

In summary, treatment of wildtype embryos during specific intervals with 800 nM of the HDAC inhibitor TSA phenocopied both the branchial arch cell-fate specification and craniofacial cartilage differentiation defects observed in *hdac1^b382^* mutants. At lower doses, milder defects in branchial arch specification and differentiation of craniofacial cartilages were observed.

### Differential Temporal Requirements for HDAC Function During Craniofacial Development

An advantage of using the pharmacological inhibitor TSA to study the effects of HDAC inhibition is the ability to identify temporal windows during development when HDAC activity is required. This was achieved for determining the temporal requirements for HDAC activity during craniofacial development by treating wildtype embryos with TSA for a fixed period of 24 h from 16, 28 and 48 hpf. After each 24 h treatment, embryos were washed clear of TSA with embryo medium and then allowed to develop until 3.5 dpf in fresh embryo medium without TSA. Embryos were then fixed and stained with alcian blue to label cartilaginous elements.

**Figure 5:**
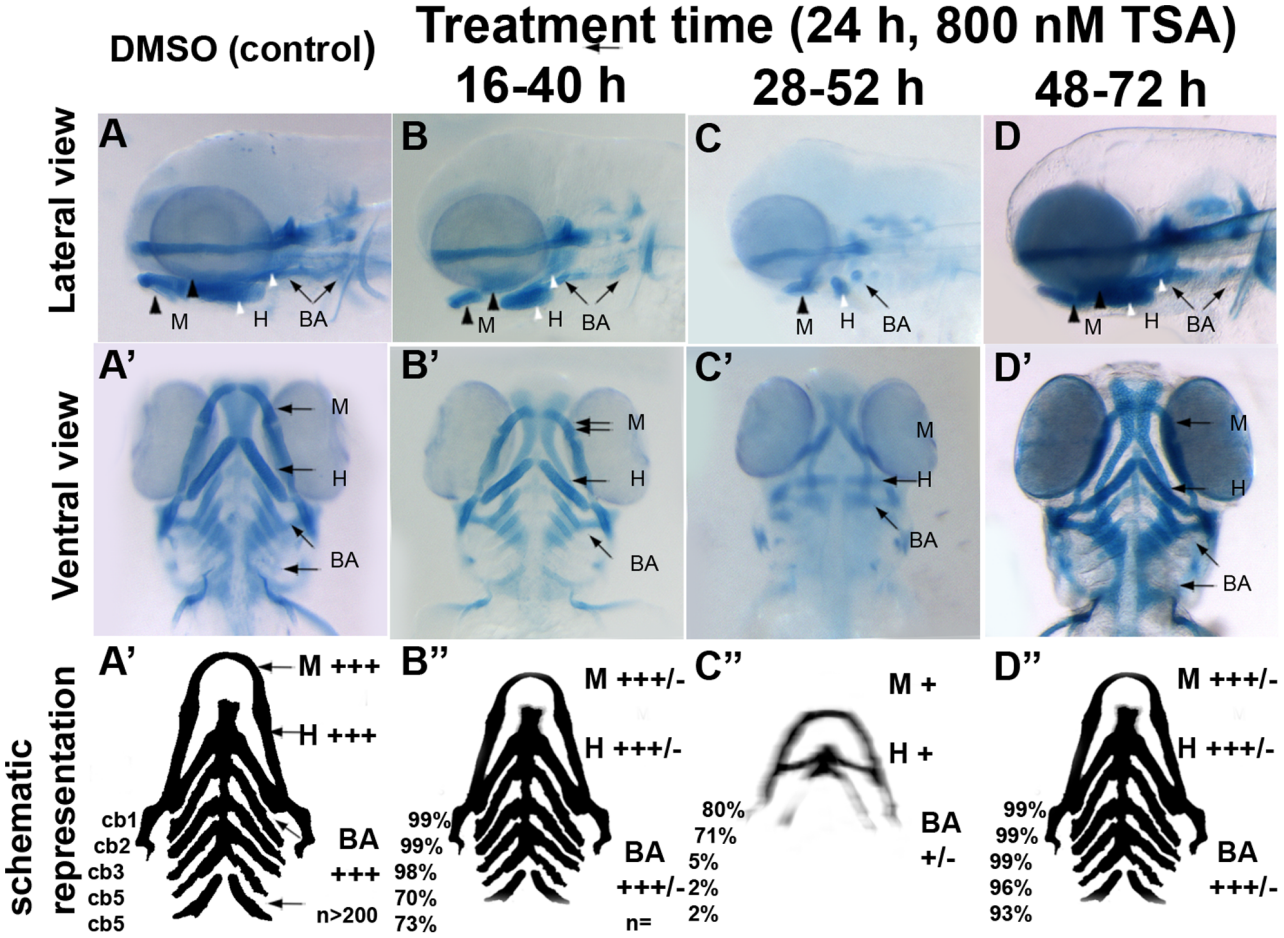
Differential temporal requirements for HDAC function during craniofacial development. A–D and A’–D’ Wild-type embryos treated with TSA for 24 hpf at different stages of embryonic development. After treatment periods other than 48–72hpf, embryos were washed to remove TSA and then allowed to develop until 3.5 dpf. Embryos were fixed at 3.5 dpf and then stained with alcian blue. A–D lateral views, A’-D’ ventral views. A–A’ are DMSO-treated controls, B–B’ 16–40 hpf TSA-treated embryos, C–C’ 28–52 hpf TSA-treated embryos, D-D’ 48–72 hpf TSA-treated embryos. A’’-D’’ schematic with summary of craniofacial defects at different TSA treatment concentrations. M, mandibular; H, hyoid; cb1-5, cerato-branchials 1-5; BA, branchial arches;+++ wild-type,++ reduced in size compared to wild-type,+Severely reduced compared to wild-type, +/− severely reduced or absent altogether.

Treatment of wildtype embryos with 800 nM TSA for 24 h between 16 and 40 hpf resulted in alcian blue stained craniofacial cartilages with reduced sized mandibular, hyoid and branchial arch elements, although the shape of the jaw in TSA treated embryos was similar to control embryos (Fig. 5 B, B’, B”; Table 2). Later HDAC inhibition with 800 nM TSA between 28 and 52 hpf resulted in further reductions in the sizes of the mandibular, hyoid and branchial arch cartilages (Fig. 5 C, C’, C”; Table 2) compared to 16–40 hpf treatments. In addition to reductions in size of all craniofacial elements, in the branchial arches, inhibition of HDAC between 28–52 hpf resulted in the complete absence of alcian blue stained ceratobranchial (cb) cartilage elements cb3, cb4 and cb5 in 95%, 98% and 98% of treated embryos (n = 161), respectively. Finally, TSA treatment of wildtype embryos between 48–72 hpf (Fig. 5 D, D’, D”; Table 2) resulted in only minor reductions in the size of all craniofacial cartilage elements when compared to control embryos and also 16–40 and 28–52 hpf TSA treatments.

**Table 2:** Temporal requirements of HDAC function during craniofacial development.

	24 h treatments with 800 nM TSA at different stages. Treated and control embryos were fixed at 3.5 dpf (84 hpf)			
	16–40 hpf	28–52 hpf	48–72 hpf	Control
Mandibular	+++/−	+	+++/−	+++
%	98.8%	86.3%	99.3%	
Hyoid	+++/−	+	+++/−	+++
%	98.8%	85%	99.3%	
CB1	++	+	+++/−	+++
%	98.8%	79.5%	98.6%	
CB2	++	+	+++/−	+++
%	98.8%	70.8%	98.6%	
CB3	++	–	+++/−	+++
%	97.7%	4.97%	97.9%	
CB4	++	–	+++/−	+++
%	70.9%	1.86%	95.9%	
CB5	++	–	+++/−	+++
%	72.7%	1.86%	92.5%	
Shape of elements	+++/−	+	+++/−	+++
N	172	161	147	

Embryos were assigned the following groups based on the severity of craniofacial malformations when compared to wild-type embryos;+++ wild-type, +++/− Mild malformation,++ Moderate malformation,+ Severe malformation, - Jaw elements absent or not stained.

Percentages reflect proportion of embryos with alcian blue stained cartilage elements to the total number of treated embryos expressed as a percentage.

Taken together, our results indicate that HDAC activity is required for distinct developmental processes during different periods of development. 800 nM TSA treatments in wildtype embryos between 16 and 40 hpf results in reduced numbers of branchial arch precursors specified. The remaining progenitor population undergoes differentiation, but with resulting reductions in the sizes of the ceratobranchials, when compared to control embryos. In contrast craniofacial cartilage differentiation in wildtype embryos is particularly sensitive to TSA treatment between 28–52 hpf, a period during which most precursors of the mandibular, hyoid and branchial arches are already specified. Also, only continuous treatment with HDAC inhibitor from 16 hpf can completely phenocopy the fate specification and differentiation craniofacial cartilage defects observed in *hdac1^b382^*mutants. Lastly, because TSA inhibition of HDAC activity closely resembles the craniofacial phenotype of *hdac1^b382^* mutant embryos, it appears that *hdac1* activity is the primary regulator of craniofacial development among the HDAC family.

### Abnormal Peripheral Sensory, Enteric and Sympathetic Neuron Development in *hdac1^b382^* Mutants

**Figure 6:**
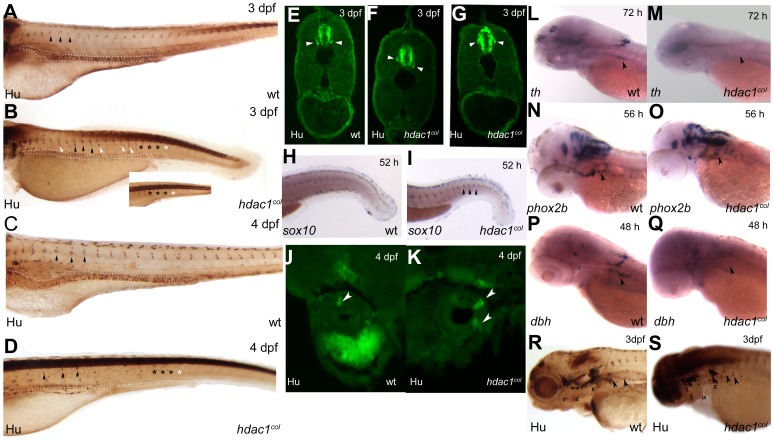
Sensory, enteric and sympathetic neuron development in *hdac1^b382^* mutants. A–D Anti-Hu/16A11 staining of wild-type and *hdac1^b382^* mutants at 3dpf and 4dpf. Black arrowheads indicate normally positioned DRG in wild-type and *hdac1^b382^* mutants. White arrowheads indicate examples of DRG neurons in ectopic locations. Black asterisks (A-D and inset) indicate the regions in the tail of wild-type and *hdac1^b382^* mutants where DRG are present. White asterisks (B and inset, D) indicate the regions in the tail of *hdac1^b382^* mutants where DRG are absent. E-G a cross sections of wild-type (E) and examples of 2 sections (F, G) of *hdac1^b382^* mutants illustrating that DRG neurons in mutant embryos are often ectopically localized. H, I wild-type and *hdac1^b382^* mutants with *sox10* expression in the tail region at 52 hpf, black arrowheads in I indicate distinct foci of *sox10* expression in mutant in locations where DRG would normally reside. J, K cross-sections of the anterior gut in wild-type and *hdac1^b382^* mutants stained with anti-Hu/16A11 labeling enteric neurons at 4 dpf (white arrowheads). L, M *th* expression in the cervical sympathetic ganglion (black arrowhead) in wild-type and *hdac1^b382^* mutants at 72 hpf. N, O, *phox2b* labeling of sympathetic neuron precursors (black arrowheads) in wild-type and *hdac1^b382^* mutants at 56 hpf. P, Q, *dbh* staining of sympathetic neurons (black arrowheads) in wild-type and *hdac1^b382^* mutants at 48 hpf. R, S, Anti Hu/16A11 staining of wild-type and *hdac1^b382^* mutants at 3dpf with black arrowheads indicating sympathetic neurons.

Neural crest cells give rise to peripheral neurons, including sensory neurons of the dorsal root ganglia (DRG), enteric neurons, and sympathetic neurons. In *hdac1^b382^* mutants, there is an overall reduction in the numbers of DRG, enteric and sympathetic neurons (Fig. 6 A–D, J, K, L–S; data not shown). In wildtype embryos at 52 hpf and 3 dpf, DRG sensory ganglia are located bilaterally adjacent to each somite pair along the length of the trunk and are positioned ventro-lateral to the spinal cord with an average of one pair of DRG neurons per ganglia as revealed by staining with pan-neuronal markers, including anti-Hu [64]. In *hdac1^b382^* mutants at 52 hpf, only 21±4 Hu^+^ DRG ganglia are present when compared to 54±4 ganglia in wildtype embryos and ganglia in mutants are preferentially present in the anterior trunk. In addition, DRG neurons of mutant ganglia are often mislocalized dorso-ventrally. Later at 3dpf in *hdac1^b382^* mutants, DRG numbers in the trunk anterior to the anus recover but very few differentiated DRG neurons are present in the tail region (Fig. 6 A–D). Also at 3 dpf, in the anterior trunk, DRG neurons in *hdac1^b382^* mutants are often present in ectopic locations when compared to wildtype embryos (Fig. 6 E–G).

Because of the absence of differentiated DRG neurons in the tail in *hdac1^b382^* mutants, we asked whether neural crest-derived cells populate nascent DRG locations in the tail of *hdac1^b382^* mutants. *sox10* is required for the specification of a subset of DRG neurons [65], and is also believed to play a role in the development of DRG-associated peripheral glia [66], [67]. Therefore we examined *sox10* expression in *hdac1^b382^* mutants as an indicator of neural crest-derived cells that contribute to the DRG. In *hdac1^b382^* mutants, *sox10* continues to be expressed robustly in potential DRG progenitors at 52 hpf and *sox10* expression is prominent in the tail region in presumptive sites of nascent DRG. In contrast, in wildtype embryos, *sox10* is no longer expressed in DRG at the same stage (Fig. 6 H, I). In contrast to differences in potential DRG progenitor *sox10*-expression, there is no difference in the expression of *sox10* in the otic vesicle between *hdac1^b382^* mutants and wildtype embryos at 52 hpf. Thus, it appears that DRG progenitors, potentially including those for DRG neurons, may be specified along the entire trunk in *hdac1^b382^* mutants, but there is a delay or failure in DRG neuron differentiation from progenitors in the tail. Since *sox10* is also expressed in neural crest derived glia which are associated with DRG sensory neurons [17], an alternative explanation for the *sox10* expression phenotype in *hdac1^b382^* mutants is that *sox10* is abnormally maintained in DRG glia irrespective of DRG neuron development and differentiation.

Enteric neurons are located along the length of the gut at 3–4 dpf in wild-type embryos [64]. In *hdac1^b382^* mutants, differentiated enteric neurons are generally absent at 3 dpf whereas the first Hu^+^ enteric neurons are observed only at 4 dpf, suggesting delayed differentiation (Fig. 6 A–D). The overall numbers of enteric neurons are also drastically reduced and they are present only in the proximal gut in *hdac1^b382^* mutants when compared to wild-type embryos, suggesting a failure in migration to, and or differentiation in, the caudal gut (Fig. 6 A–D data not shown).

Differentiated sympathetic neurons utilize noradrenalin as a neurotransmitter and therefore express the catecholamine biosynthetic enzymes *tyrosine hydroxylase* (*th*) and *dopamine ß-hydroxylase* (*dbh*) [68], [69]. Sympathetic neurons in *hdac1^b382^* mutants fail to express *th* or *dbh* at 2 and 3 dpf suggesting that they do not undergo noradrenergic differentiation (Fig. 6 L, M. P, Q; data not shown). Nonetheless, in *hdac1^b382^* mutants at 2 dpf, sympathetic neuron precursors are present as diagnosed by robust expression of the transcription factors *phox2b* and *zash1a*, genes that are expressed by and required for sympathetic neuron precursor development [68], [69] (Fig. 6 N, O; data not shown). We also analyzed the expression of *hand2,* a gene that is required for sympathetic neuron noradrenergic differentiation [70]. In *hdac1^b382^* mutants at 2 dpf, *hand2* is expressed in only a few sympathetic neuron precursors as compared to the far greater numbers of sympathetic neuron precursors which express *hand2* in wildtype embryos (data not shown). At 3 dpf in *hdac1^b382^* mutants, Hu^+^sympathetic neurons are present (Fig. 6 R, S), indicating the differentiation of some sympathetic precursors into neurons. Thus, analysis of sympathetic neuron gene expression reveals that in *hdac1^b382^* mutants sympathoadrenergic precursors are specified and many undergo overt neuronal differentiation but that the noradrenergic differentiation of sympathetic neurons fails to occur.

In summary, there is an overall delay in the differentiation of neural crest-derived DRG, enteric and sympathetic neurons of the peripheral nervous system in *hdac1^b382^* mutants. Enteric and DRG neurons in the tail are the most severely affected while the DRG and enteric neurons in the anterior trunk are less affected in *hdac1^b382^* mutant embryos. In addition, whereas the specification and neuronal differentiation of sympathetic neurons is largely normal, noradrenergic differentiation fails to occur in *hdac1^b382^* mutants.

### Blockade of the Noradrenergic Differentiation of Sympathetic Neurons by Reduced *hdac1*/HDAC Activity is Reversible

**Figure 7:**
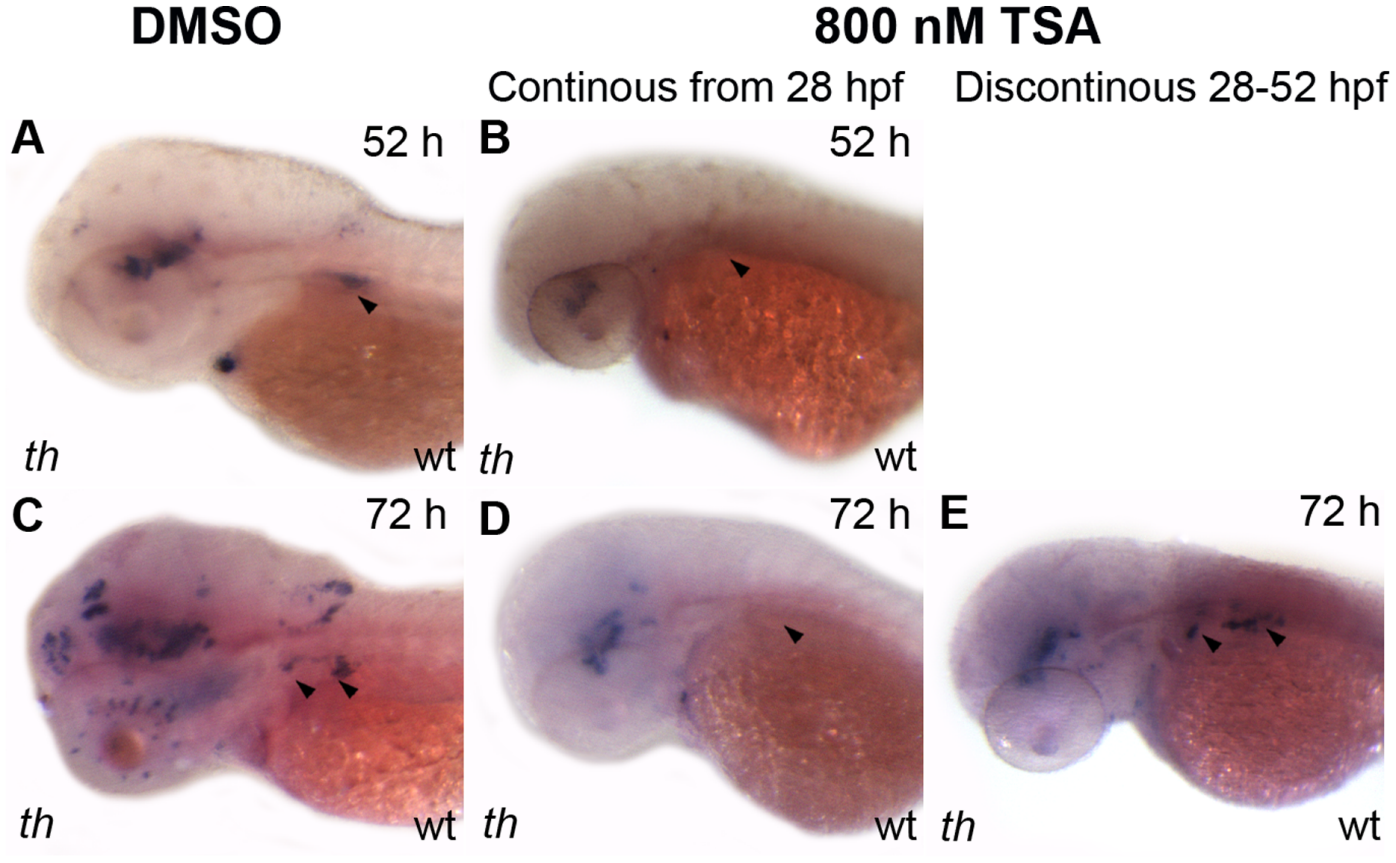
Effect of HDAC inhibition on sympathetic neuron differentiation is reversible. A, B wild-type embryos treated with DMSO (A) and TSA (B) continuously from 28–52 hpf and then fixed at 52 hpf and stained for *th* expression, black arrowheads indicate sympathetic neurons. C–E wild-type embryos treated under three different conditions, C with DMSO between 28–72 hpf, D with TSA between 28–72 hpf, and E with TSA between 28–52 hpf after which the TSA is washed out and then the embryos are treated in DMSO between 52–72 hpf. All embryos were fixed at 72 hpf and then stained for *th* expression. Black arrowheads indicate *th*-xpressing sympathetic neurons, or their absence.

Disruption of Hdac1 function in *hdac1^b382^* mutants results in the failure of the noradrenergic differentiation of sympathetic neurons based on the absence of *th* and *dbh* expression even though sympathoadrenergic precursors cells are present, and many of which undergo generic neuronal differentiation. We therefore assessed the effect of TSA-induced HDAC inhibition on noradrenergic differentiation of sympathetic neurons by testing the effect of 800 nM TSA on *th* expression in sympathetic neurons (Table 3). Treatment of wildtype embryos for 24 h between 28–52 hpf results in the complete absence of *th*-expression in sympathetic ganglia (0%, n = 82, Fig. 7 A, B). In contrast to sympathetic neuron *th*-expression, TSA-treated embryos retain *th*-expression in non-neural crest-derived midbrain dopaminergic neurons and arch associated ganglia (100% and 98.8% of treated embryos respectively, n = 82). All wildtype control (DMSO treated) embryos expressed *th* in the sympathetic ganglia, midbrain dopaminergic neurons and arch associated ganglia (n = 140). In *hdac1^b382^* mutants, sympathetic neuron precursors express *phox2b* and *zash1a*. We therefore analyzed *phox2b* expression in 28–52 hpf 800 nM TSA-treated embryos. 95% (n = 78) of all TSA treated wildtype embryos express *phox2b* in the sympathetic ganglia at 52 hpf, even though qualitatively, the overall ganglia size is slightly reduced when compared to control DMSO-treated wild-type embryos (Figure S1). These data indicate that treatment of wild-type embryos for 24 h between 28–52 hpf results in defects in noradrenergic differentiation of sympathetic neurons similar to *hdac1^b382^* mutants. These results also indicate that the effect of HDAC inhibition on *th* expression is cell type specific with midbrain dopaminergic neurons and arch associated ganglia *th*-expression relatively unaffected by TSA, compared to neural crest-derived sympathetic neurons.

**Table 3:** Effect of 800 nM of TSA on sympathetic neuron *th* expression.

	800 nM TSA treatment for24 h and fixed at 52 hpf		800 nM TSA treatment for 24/48 h andfixed at 72 hpf		
Time of treatment	TSA 28–52 hpf	Control 28–52 hpf	Treated 28–52 hpf	Treated 28–72 hpf	Control 28–72 hpf
Sympathetic neurons	0%	100%	96%	13.7%	100%
Locus coreleus	12.2%	99.3%	93%	54.8%	100%
Arch associated cells	98.8%	100%	96%	86.3%	100%
Mid-brain dopaminergic neurons	100%	100%	89%	77.4%	100%
Negative (unstained embryos)	0/82	0/140	1/100	4/124	0/125
N	82	140	100	124	125

Percentages reflect the proportion of embryos with *th*-expression to the total number of treated embryos expressed as a percentage.

We then tested whether the TSA-induced failure of noradrenergic differentiation of sympathetic neurons is reversible. To address this question, we treated wild-type embryos with 800 nM of TSA for 24 h from 28–52 hpf. Treated embryos were then washed with embryo medium to remove TSA and allowed to develop until 72 hpf in embryo medium without TSA. At 72 hpf, embryos were fixed and analyzed for *th* expression. 96% (n = 100) of 28–52 hpf TSA-treated wild-type embryos had *th*-positive sympathetic neurons present at 72 hpf when compared to only 14% (n = 124) of embryos with *th*-positive sympathetic neurons that were treated continuously between 28–72 hpf with TSA (Fig. 7 C-E; Table 3). This indicates that the inhibition of noradrenergic differentiation by the disruption of Hdac1/HDAC activity is reversible if the inhibition of HDACs is relieved.

### Hdac1 Levels are Progressively Reduced in *hdac1^b382^* Mutants

**Figure 8:**
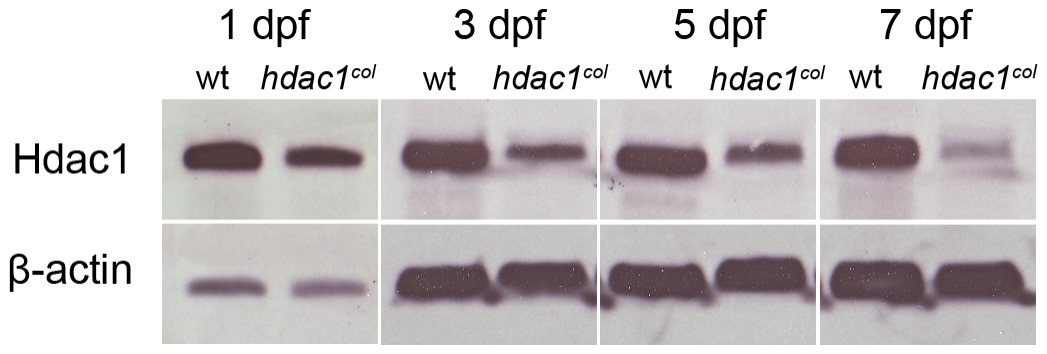
Hdac1 levels are severely reduced in *hdac1^b382^* mutants. Western blots for total Hdac1 from wild-type and *hdac1^b382^* (labeled *hdac1^col^*) mutants at 1, 3, 5 and 7 dpf. ß-actin was used as a loading control.

A feature of the *hdac1^b382^* mutant phenotype is the progressive perturbation of the development of multiple tissues over time culminating with death by 7 to 8 dpf. While there are clear gene expression patterning defects as early as gastrulation, obvious visible differences between mutants and wild-type embryos are first observed at 27 hpf highlighted by pigmentation abnormalities and then at 48 hpf by cardiac, pectoral fin and jaw defects [33]. The progressive elaboration of developmental defects in mutant embryos could be due to cumulative effects of constant relative reduced levels of Hdac1 protein or potentially to a progressive reduction in relative Hdac1 levels. We therefore analyzed the levels of Hdac1 protein in wildtype and mutant embryos at 1, 3, 5 and 7 dpf by Western analysis (Fig. 8). We find that Hdac1 protein levels are reduced in mutants when compared to wild-type embryos at all time points analyzed. Further, as development progresses, whereas Hdac1 levels in wildtype embryos and larvae increase, relative Hdac1 levels decrease in mutant embryos such that by 7 dpf, Hdac1 protein is not readily detectable in mutant embryos. These results raise the possibility that the progressive nature of the *hdac1^b382^* mutant phenotype results from decreasing relative levels of Hdac1 protein as development proceeds, possibly due to an autoregulatory mechanism controlling Hdac1 expression or due to the degradation of maternally supplied mRNA/protein over developmental time.

## Discussion

The regulation of the acetylation states of proteins, prominently that of histones, is mediated by the antagonistic functions of the two large families of enzymes composed of histone acetyltransferases (HATs) and histone deacetylases [71]. The consequence of histone acetylation/deacetylation on gene expression is largely thought to be that increased acetylation promotes gene expression whereas deacetylation inhibits expression, although exceptions to this general rule have been identified. Further, the acetylation of proteins other that histones has been shown to be modulated by HATs and HDACs, although the functional consequences of these modifications are not well understood. Overall, the regulation of histone acetylation state is considered to be a significant regulator of gene expression. To be further resolved is the extent to which the functions of different members of the two large families of enzymes mediate the expression of limited and specific subsets of genes regulating discreet processes during development as opposed to more global gene expression patterns.

Our results of phenotypic analysis of *hdac1^b382^* mutants and the selective temporal manipulation of HDAC function during development with the HDAC inhibitor TSA demonstrate a reiterative requirement for *hdac1* function during craniofacial neural crest development, including the specification of neural crest posterior branchial arch progenitors, cranial neural crest cell migration and morphogenesis and, ultimately, the differentiation of all neural crest-derived craniofacial cartilage elements. Specifically, abrogation of Hdac1 function resulting from significantly reduced Hdac1 protein levels in *hdac1^b382^* mutants causes defects in the specification of subpopulations of posterior cranial neural crest progenitors and results in the subsequent failure in the morphogenesis and differentiation of posterior pharyngeal arch elements. In contrast, anterior branchial arch progenitors are specified normally in *hdac1^b382^* mutants, but their migration, morphogenesis and differentiation depends on normal Hdac1 function. Thus, our results demonstrate that during craniofacial neural crest development *hdac1* is required at different times and for distinct developmental processes for cranial neural crest subpopulations at different anterior-posterior levels.

Our observations concerning the overall craniofacial phenotype of *hdac1^b382^* mutants is reminiscent of that previously reported in *hdac1* morphants and the insertional mutant allele *hi1618* [37]. Specifically, we report early defects in cranial neural crest *dlx2* expression, particularly in posterior arches and a comparable abnormal expression pattern was observed for *dlx4* by Pillai et al., 2004. Likewise, similar prominent defects in the development of cartilaginous elements in the head detected by alcian blue staining were observed, although the overall severity of defects observed was different with respect to anterior elements, possibly due to either the analysis of embryos with different levels of functional *hdac1* abrogation and/or differences in the stages of development examined. Nevertheless, overall, the similarities in these observations strongly indicate a critical role for normal *hdac1* function for the proper development of the neural crest-derived craniofacial cartilages in zebrafish.

Our results identify and detail specific asynchronous and functionally distinct requirements for *hdac1* function during the development of a discreet embryonic population, the neural crest, in contrast to the comparatively more recognized global functions of Hdacs in the regulation of gene expression, and as such illustrate the developmental heterogeneity within the developing early neural crest cell population. In a broad context, the necessity for *hdac1* function is differentially required for distinct developmental processes between cranial and trunk neural crest populations. Further, within these subdivisions, *hdac1* is further asymmetrically deployed. Most dramatically, within the cranial neural crest population, as described above, *hdac1* function is required aynchronously and for developmentally distinct, essential processes for subpopulations of progenitors present at different axial levels. These results support the idea that early developmental heterogeneity likely exists between topographically distinct cranial neural crest populations [57], [72], [73], and our results clearly demonstrate that the developmental mechanism and timing requirements for *hdac1* activity between cranial neural crest cells at different axial levels is distinct and raises the possibility that this differential requirement for *hdac1* function may contribute to the establishment and/or elaboration of early heterogeneity within the overall cranial neural crest population.

The requirement for *hdac1* function is also mechanistically heterogeneous for the development of trunk neural crest. We previously showed that *hdac1* function in necessary for the repression of neural crest *foxd3* expression permitting *mitfa*-dependent melanogenesis [32]. Thus, *hdac1* is normally required for the specification of melanogenic precursors, mechanistically similar to the requirement for *hdac1* by posterior arch progenitors in the head. Here we show that reduced *hdac1* function results in the temporal retardation of the migration and differentiation of both enteric and dorsal root ganglion sensory neuron progenitors. This requirement for *hdac1* in the trunk neural crest appears mechanistically similar to that for *hdac1* among anterior pharyngeal arch cranial neural crest cells.

The differential requirements for *hdac1* activity within the overall neural crest lineage is further illustrated by the very specific requirement for appropriate *hdac1* activity during the development of sympathetic neurons. We found that the specification of sympathetic neuron progenitors (sympathoblasts), their migration to the vicinity of the dorsal aoarta and their neuronal differentiation does not require normal levels of *hdac1* expression and/or activity as determined in *hdac1^b382^* mutants and TSA-treated wildtype embryos. In contrast, normal Hdac1 expression levels and activity are required for the expression of the biosynthetic enzymes DßH and TH by neural crest-derived sympathoblasts and thus the establishment of the noradrenergic phenotype of sympathetic neurons. Interestingly, the results of our experiments transiently inhibiting Hdac activity with the application of TSA to wildtype embryos illustrates that the noradrenergic differentiation of sympathetic neurons in zebrafish is temporally quite plastic as the expression of TH and DßH can successfully occur in sympathetic neurons even after extended Hdac activity blockade.

Based on our results with mutant embryos and in experiments with temporally controlled pharmacological blockade, we favor the interpretation of heterogeneous requirements for Hdac1 activity by different populations of neural crest cells at different stages of development and for different processes. Nevertheless, based on our analysis of Hdac1 protein levels (Fig. 8) it is also possible that the decreased levels of Hdac1 protein between 1 and 3 dpf may also play a role in the observed phenotypes. On the other hand, we also do not know if any of the Hdac1 protein detected in mutant embryos is functional and if not, whether functional mRNA/protein is maternally supplied and relatively stable, diminishing only after several days of embryogenesis. In any case, to resolve these uncertainties it will be necessary to directly measure Hdac1 activity in future studies.

In this study we show that *hdac1^col^* is required for the differentiation of multiple neural crest derivatives including craniofacial cartilages and neurons of the peripheral nervous system. Previous studies in *hdac1* mutants have identified a requirement for *hdac1* in the differentiation of melanophores, the eye, and neurons and oligodendocytes in the CNS [31], [32], [34], [35], [74]. Thus, an emerging common function of *hdac1* during zebrafish embryonic development appears to be in regulating cell fate specification and cellular differentiation. Consistent with this proposed function, in *hdac1^col^* mutants neural crest cells robustly express precursor genes *foxd3* and *sox10* well after they are downregulated in wild-type embryos, indicating a delay in differentiation of at least some neural crest precursors [32]. Similarly, in the CNS, there is failure to downregulate the Notch signaling pathway, which is usually expressed in undifferentiated neurons [31]. The function of *hdac1* in embryonic cell diversification that we propose is in contrast to the requirement of HDAC inhibitors in promoting differentiation. Specifically, HDAC inhibitors have been approved in the treatment of cutaneous T-cell lymphoma, where HDAC inhibitors drive the terminal differentiation of cancer cells [30]. Also, TSA has been used extensively in many studies to increase the differentiation of multiple cell types [30], [40], [41]. These seemingly conflicting functions of *hdac1*/Hdacs could be due to different cellular contexts and/or changes in functional requirements with temporal alterations in the regulation of gene expression between embryonic and more mature and cancer cell types. Further, the precise levels of *hdac1*/Hdac expression and activity also appear to be important in determining function.

In summary, our results further uncover the specific requirements for *hdac1* function during the development of the neural crest in zebrafish. One of the future challenges will be to identify the gene targets of *hdac1* regulation during neural crest development and whether regulation occurs at the level of histone acetylation states and/or other *hdac1*-regulated modifications. The existence of multiple zebrafish mutant alleles, if they differ in the extent of disruption of Hdac1 function, which is not formally known currently, may provide powerful tools, along with other experimental approaches, toward further understanding *hdac1* function during embryogenesis.

## Supporting Information

Figure S1 **Effect of TSA on sympathetic neuron development.** A, C wild-type embryos treated with DMSO, B, D wild-type embryos treated with TSA continuously from 28–52 hpf and 28–72 hpf and then fixed at 52 hpf and 72 hpf stained for *phox2b* expression, black arrowheads indicate sympathetic neurons.
(TIFF)
