# Supplementary figures and images for: Distinct Functional and Temporal Requirements for Zebrafish Hdac1 during Neural Crest-Derived Craniofacial and Peripheral Neuron Development
Source: PLoS One. 2013 May 7;8(5):e63218. doi: 10.1371/journal.pone.0063218 (PMC3646935; doi:10.1371/journal.pone.0063218)

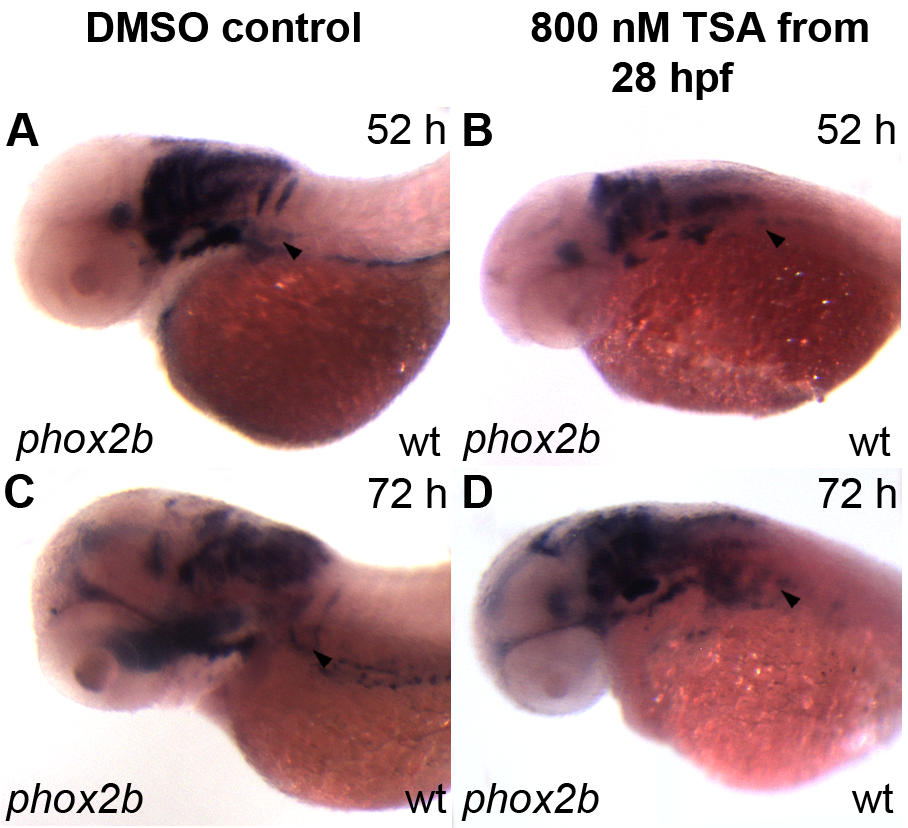

Supplement: Figure S1 — Effect of TSA on sympathetic neuron development. A, C wild-type embryos treated with DMSO, B, D wild-type embryos treated with TSA continuously from 28–52 hpf and 28–72 hpf and then fixed at 52 hpf and 72 hpf stained for phox2b expression, black arrowheads indicate sympathetic neurons. (TIFF) [file pone.0063218.s001.tiff]
